# Statistics of Mortality

**Published:** 1873-02-01

**Authors:** 


### MEDICAL EXAMINER.

*A Semi-Monthly Journal of Medical Sciences.*

EDITED BY

N. S. DAVIS, M. D., AND F. H. DAVIS, M. D.

Chicago, February 1st, 1873.

### EDITORIAL.

### STATISTICS OF MORTALITY.

From an abstract of the annual report of the Sanitary Superintendent of this city, contained in one of our daily papers, we learn that the total number of deaths in Chicago during the year 1872 was 10,157. This is an increase of 3,181 over the number of deaths in 1871, and 2,814 over the number for 1870. The following table shows the amount of sewerage, the total population, the total mortality, and the ratio of deaths to each 1,000 of the population for a period of seventeen years:

| YEAR. | Feet of sewers constructed. | Population. | Mortality. | Death rate per 1,000. |
|---|---|---|---|---|
| 1856 | 31,794 | 84,113 | 2,086 | 24.80 |
| 1857 | 25,681 | 93,000 | 2,414 | 25.66 |
| 1858 | 101,879 | 84,000 | 2,255 | 26.84 |
| 1859 | 55,208 | 96,000 | 2,008 | 21.36 |
| 1860 | 69,024 | 109,260 | 2,264 | 20.70 |
| 1861 | 2,826 | 120,000 | 2,279 | 18.99 |
| 1862 | 15,685 | 137,030 | 2,835 | 20.69 |
| 1863 | 39,605 | 150,000 | 3,875 | 25.83 |
| 1864 | 25,021 | 161,288 | 4,448 | 27.57 |
| 1865 | 29,948 | 178,492 | 4,029 | 22.57 |
| 1866* | 48,127 | 200,418 | 6,524 | 32.22 |
| 1867 | 89,661 | 225,000 | 5,648 | 21.17 |
| 1868 | 47,841 | 252,054 | 5,984 | 23.74 |
| 1869 | 139,705 | 280,000 | 6,488 | 23.16 |
| 1870 | 78,166 | 299,227 | 7,343 | 24.53 |
| 1871 | 50,392 | 325,000 | 6,976 | 21.46 |
| 1872† | 42,767 | 367,293 | 10,157 | 27.60 |

The following table will show the connection between the rain-fall for the three warmest months, the total for the year, and the ratio of deaths to population, for a period of seven years:

* Cholera year. † Small-pox, 654.

| YEAR. | July, inches. | Aug., inches. | Sept., inches. | Total for three months. | Total, year. | Death rate per 1,000. |
|---|---|---|---|---|---|---|
| 1866 | 3.58 | 7.84 | 6.53 | 17.95 | 36.65 | 22.32 |
| 1867 | 1.51 | 2.32 | 0.40 | 4.23 | 21.26 | 21.16 |
| 1868 | 3.86 | 3.58 | 7.08 | 14.52 | 37.33 | 23.74 |
| 1869 | 3.21 | 1.38 | 0.89 | 5.48 | 31.66 | 23.16 |
| 1870 | 3.71 | 2.17 | 2.82 | 8.70 | 23.62 | 24.53 |
| 1871 | 2.56 | 0.50 | 0.10 | 3.16 | 32.85 | 21.46 |
| 1872 | 4.05 | 2.56 | 6.43 | 13.04 | 28.94 | 27.61 |

The connection between the temperature of the four warmest months and the death rate is shown by the following table, representing the same years as the preceding:

| YEAR. | MEAN DAILY TEMPERATURE. | | | | Mean for four months, deg. | Death rate for year in 1,000. |
|---|---|---|---|---|---|---|
| | June, deg. | July, deg. | Aug., deg. | Sept., deg. | | |
| 1866 | 70 | 70 | 71 | 64 | 68.3 | 32.22 |
| 1867 | 72.4 | 72.9 | 72.8 | 66 | 71 | 21.17 |
| 1868 | 66.3 | 81.4 | 73.3 | 63.2 | 71 | 23.74 |
| 1869 | 65.4 | 73.8 | 73.5 | 69.5 | 70 | 23.16 |
| 1870 | 70.2 | 79.6 | 75 | 71 | 73.3 | 24.53 |
| 1871 | 72 | 72.3 | 71.3 | 67 | 70.2 | 21.46 |
| 1872 | 70.8 | 72.4 | 72 | 64 | 69.2 | 27.60 |

Of the 10,157 deaths in 1872, no less than 6,127 are reported as children under six years of age. Of the latter, 493 died in the month of June, 1,059 in July, 1,137 in August, and 553 in September—making considerably more than half of the whole number in the four months named. A further examination of the figures, shows that the ratio of deaths under five years of age, during the four months last named, was double among children of foreign parentage that occurring among children of native parentage.

The chief causes of death among the children under five years of age, for six months of the year (from April 1st to September 30th), together with the rain fall and the mean temperature, are shown in the following table :

| CAUSES. | April. | May. | June. | July. | August. | September | Total. |
|---|---|---|---|---|---|---|---|
| Chol. infantum | 6 | 10 | 146 | 549 | 530 | 187 | 1,428 |
| Convulsions | 92 | 71 | 161 | 112 | 116 | 81 | 573 |
| Diarrhœa | 8 | 5 | 53 | 73 | 132 | 63 | 384 |
| Dysentery | 2 | 3 | 26 | 38 | 63 | 30 | 162 |
| Tabes mesenterica | 8 | 14 | 21 | 40 | 38 | 27 | 148 |
| Teething | 3 | 14 | 16 | 25 | 34 | 14 | 106 |
| Total | 119 | 117 | 463 | 837 | 913 | 402 | 2,801 |
| Mean daily temperature | .... | 57½ | 70⅛ | 72½ | 70 | 64 | .... |
| Rain fall | 299 | 329 | 341 | 408 | 256 | 643 | .... |

Nearly all of those ascribed to "teething," in this table, should have been added to the list of bowel affections. Teething is a natural process, and we have never yet seen a child die simply from the growth of its teeth; and we think it is quite time that physicians should cease to use the word to designate the cause of death in their certificates sent to the office of the Board of Health. If the Sanitary Superintendent is correctly reported, he gives a large amount of attention to the subject of sewerage; and by a detailed statement of the amount of sewerage in each ward, in connection with the population and ratio of deaths, he makes it appear that the death rate decreases in direct ratio to the increase of sewerage. He makes this a basis for strongly urging upon the city authorities the necessity for a more rapid extension of sewerage system. All this is right, so far as it bears on the sanitary benefit to be derived from good sewerage. And yet those who are familiar with the different wards of the city, will readily see that the same wards that have the least amount of sewerage also have the largest proportion of foreign population; a fact that greatly influences the ratio of mortality, as shown in that part of the Superintendent's report relating to infantile mortality.

It was there shown that the ratio of deaths of children under five years of age, born of foreign parentage, was more than double that of children born of native parentage. Our own observation has long since taught us that this ratio holds good when the native and the foreign parents occupy the same ward; and hence we must look for other causes besides sewerage to explain a part, at least, of the results. We do not care to diminish the force of the Superintendent's argument in favor of sewerage; but, while touching on this subject, we are constrained to express the opinion that sewers are of very little value, in a sanitary point of view, unless they are properly connected by side-drains with the adjacent lots and buildings, and such side-drains and sewer-entrances are kept free from obstructions. And truth compels us to add, that negligence, and want of system in attending to this part of the subject, has thus far deprived us of half the benefit that ought to have been derived from the sewerage already constructed. Every year we have seen whole blocks saturated with surface water until the middle of June, and sometimes until July, when there were good sewers within reach; and three days' work, with hoe and shovel, in cleaning side-ditches, would have drained any one of them before the first of April, and left the subsequent spring rains to have washed and cleansed the surface, instead of mascerating until evaporated by a summer's sun. Let our Board of Health get as much sewerage constructed as they can; but if they will cause what we have to be properly connected with the adjacent lots, put on a sufficient force early every spring to thoroughly open all the sewer entrances, ditches, and side-drains, and exchange their special sanitary police for an equal force of intelligent laborers, armed each with hoe and shovel, to act as sanitary patrolmen, in keeping everything connected with the sewers free from obstructions, they will make a greater improvement in the sanitary condition of the city than by any other process.

